# Psychometric properties of the Seville quality of life questionnaire in Mexican patients with psychosis

**DOI:** 10.1186/s12888-016-0877-7

**Published:** 2016-05-25

**Authors:** Lizzette Gómez-de-Regil

**Affiliations:** Hospital Regional de Alta Especialidad de la Península de Yucatán (HRAEPY), Calle 7, No. 433 por 20 y 22, Fraccionamiento Altabrisa, Mérida, Yucatán 97130 Mexico

**Keywords:** Seville quality of life questionnaire, Schizophrenia, Psychosis, Quality of life, Mexico

## Abstract

**Background:**

The Seville Quality of Life Questionnaire (CSCV) was designed to assess quality of life in patients with schizophrenia taking into account those facets particularly important in this disorder. The study aimed at applying the CSCV to a sample of Mexican patients with psychosis in an effort further substantiates the psychometric properties of the CSCV.

**Methods:**

This cross-sectional study included 61 patients (56 % female) with psychosis. Item-scale and item-factor correlations were analyzed, as well as scale-factor correlations. Cronbach’s Alpha and principal component analysis with varimax rotation were used to assess internal consistency and construct validity, respectively.

**Results:**

Analyses of both, disfavorable and favorable dimensions, showed a stronger item-scale than item-factor correlation, in most cases significant, though. Internal consistency was significant and adequate; higher for scales than for factors. For the disfavorable and the favorable scales 11 and 2 factors were obtained, respectively; explained variance was low.

**Conclusions:**

In this sample of Mexican patients it was replicated that the CSCV is a valid and reliable instrument to assess quality of life in people with psychosis; the use of scale scores is recommended.

## Background

Paradigms in the assessment of human health have been changing for decades. Human health is currently defined as a state of complete physical, mental and social well-being, rather than the mere absence of disease or illness [1]. Assessment of a patient’s health is no longer based solely on a clinician’s report of the illness, but now includes the patients’ appraisal of their condition as a vital input [2]. An intrinsic element of this new perspective of health is quality of life (QoL), defined as the individuals’ perception of their positions in life in the context of the culture and value systems in which they live and in relation to their goals, expectations, standards and concerns [3]. The concept of QoL is increasingly acknowledged as an important health index and has garnered greater attention in both clinical practice and research.

Growing interest in the study of QoL in the healthcare community has been driven by recognition of health’s subjective dimension, as well as the increasing prevalence of chronic illnesses that require long-term, and in some cases permanent, treatment. Greater numbers of patients now receive adequate treatment that allows them to control their symptoms, and even survive illnesses that were previously treated as terminal. However, this has created the challenge of procuring patient QoL in addition to curing or controlling their ailment [2].

Mental health care must also face this challenge. Traditionally, symptom presence and severity, patient functional status and the adverse effects of medication have been the most widely used outcome metrics in psychiatric care. All are usually reported by clinical staff (psychiatrists, psychologists, nurses, social workers) and/or relatives, supporting the prevailing perspective that self-reporting by psychiatric patients is unreliable [4]. However, advances in both pharmacological and psychotherapeutic treatments have significantly improved patients’ observed clinical status, making them more qualified as informants of the subjective experience of their disorders.

Conceptual and methodological debate is still active in this area [5, 6], but there is growing interest in the study of QoL in psychotic patients [7–12]. Most studies report on use of generic QoL questionnaires that are useful to a degree, but may not comprehensively reflect the particular experience of schizophrenia when comparing diverse samples (i.e. from the general population or patients with disorders other than psychosis). For schizophrenic patients, QoL depends on myriad factors, including cultural and economic circumstances, societal homogeneity, traditional role of the family, social support, and availability of mental health services [12]. A consistent and negative association has been identified between psychopathology and QoL [7], yet there is no reason to consider the presence of symptoms in psychiatric patients as defining their QoL status. The possible side effects (e.g. lethargy, fatigue) of the antipsychotics used to control positive psychotic symptoms also need to be accounted for since they can negatively impact QoL [13, 14].

In response to this situation, a group of experts in Spain developed the Seville Quality of Life Questionnaire (Cuestionario Sevilla de Calidad de Vida - CSCV) to explore crucial aspects of QoL in schizophrenic patients. Their initial item set consisted of 126 items, but this was reduced to 59 in the final instrument following factorial analyses. The CSCV includes a disfavorable scale (CSCV-D) with 46 items, and a favorable scale (CSCV-F) with 13 items [13–16]. Accurate psychometric properties have been reported for this instrument in samples from Spain [15] and Chile [17]. It has been applied in samples from Spain [18–21], Chile [22–25], Peru [26], and Mexico [27]. Although it is targeted at patients with schizophrenia and related psychosis, it has also been found useful for patients with cases of severe drug addiction [26].

The present study’s aim was to apply the CSCV to a sample of Mexican patients with psychosis in an effort further substantiate the psychometric properties of the CSCV. A reliable and valid instrument is vital to assessing QoL in psychotic patients because it helps to generate dependable research data that can be used to design, implement and evaluate new clinical intervention strategies. Previous studies suggest the CSCV as an effective diagnostic option, particularly, but not limitedly, for Spanish-speaking countries such as Mexico, but its psychometric properties need to be confirmed in target populations before it is applied widely.

## Methods

### Design and participants

Partial data were used from a cross-sectional study on the QoL of psychotic patients and their primary caregivers. The study was done in 2009 at the Yucatan Psychiatric Hospital (Hospital Psiquiátrico Yucatán) in Merida, Mexico. This is the only public institution in the city offering specialized psychiatric care. Following Declaration of Helsinki norms [28], the study protocol was approved by the Bioethics Committee of the host hospital. Patient inclusion criteria were: 1) age at onset 16–45 years; 2) primary current DSM-IV-TR [29] diagnosis of schizophrenia or other schizophrenia spectrum psychotic disorder; and 3) inhabitant of Merida. Exclusion criteria were: 1) a DSM-IV-TR diagnosis of affective, organic, or toxic psychosis [29]; 2) evident intellectual disorder; 3) inadequate contact information; and 4) severe symptomatology impeding patient self-reporting.

A review of clinical files according to these criteria produced 161 potential cases; of which 103 patients could be contacted (55 had moved away or were not home, 3 had died). Five of the remaining candidates were excluded due to severe symptomatology. The final sample included 61 participants (56 % female) who signed an informed consent form including a statement of no economic compensation. None of the participants was hospitalized.

### Sample characteristics

Mean age of patients was 35.9 years (SD = 10.0) when interviewed, and 29.1 years (SD = 9.8) at psychosis onset. No differences by sex were observed. Thirty-four (56 %) participants had an education level of middle school or lower (up to 9^th^ grade), and the remaining 27 (44 %) had a partial/complete high school or above education level. The DSM-IV-TR diagnoses for participants were 41 patients with schizophrenia (14 paranoid, 2 disorganized, and 25 residual); and 20 patients with other types of schizophrenia-spectrum psychoses (8 schizoaffective, 7 delusional, 2 schizophreniform, 2 brief, and 1 unspecified). Mean illness course was 6.7 years (SD = 1.9, range 3.8–11.2).

Clinical status was measured using the PANSS (Positive and Negative Syndrome Scale) [30], with possible scores ranging from 1 (absent) to 7 (extreme). Mean scores in the sample were 1.44 (SD = .52) for positive symptoms, 1.67 (SD = .75) for negative symptoms and 1.55 (SD = .42) for the general psychopathology dimension. Mean GAF (Global Assessment of Functioning) [29] score for the sample was 75.3 (SD = 16.5). No differences by sex were identified for any of the PANSS or GAF scores.

Overall mean score for the CSCV-D was 1.71 (SD = .60, range 1.00–3.37), with mean scores for the corresponding nine factors falling within a range of 1.60 to 1.85. Overall mean score for the CSCV-F was 3.05 (SD = .68, range 1.54–4.00), and mean scores for the corresponding three factors ranged from 2.89 to 3.15. No significant differences were identified by sex when comparing the overall CSCV-D and CSCV-F scores, and neither were differences found between any of their corresponding factors.

### Instruments

The CSCV [14, 15] includes 59 Likert scale items, ranging from 1 (totally disagree) to 5 (totally agree). The first 46 items correspond to the CSCV-D scale and are grouped into nine factors; they refer to unpleasant, negative or unsatisfactory aspects (e.g. “I’m bored all the time”, “I’m afraid of myself”, “Everything overwhelms me”). The last 13 items, grouped in three factors, correspond to the CSCV-F scale, and reflect pleasant, positive and satisfactory aspects (e.g. “I like myself”, “I feel comfortable with my thoughts”, “I am capable of organizing my daily life”). The dimensions and their corresponding factors are listed in Table 1. Mean scores can be obtained for the 12 factors and the 2 subscales by averaging their corresponding items. Scores of 1 on the CSCV-D and 5 on the CSCV-F reflect a significantly high quality of life, with eventual psychopathological problems having no or only minimal impact. Conversely, scores of 5 on the CSCV-D and 1 on the CSCV-F represent a patient who estimates his/her QoL as extremely unfavorable or negative, and considers aspects prototypical of good QoL to be absent. A mean CSCV-D score between 3 and 5 identifies patients whose QoL is negatively affected, regardless of their CSCV-F score [13].

**Table 1:** Item-factor and item-scale Spearman correlations (item included in total score/item excluded in total score)

Disfavorable Scale (CSCV-D)		
Factor	Item – factor correlation	Item – scale correlation
Item number	r_s_	r_s_
Lack of cognitive apprehension	$\overline{x}=.657/.485$	$\overline{x}=.515/.496$
1	.615/.522	.352/.334
2	.870/.702	.640/.619
3	.757/.589	.527/.503
5	.574/.363	.606/.591
35	.468/.250	.452/.434
Loss of energy	$\overline{x}=.680/.604$	$\overline{x}=.627/.609$
4	.647/.579	.593/.571
9	.760/.653	.638/.625
10	.744/.681	.613/.592
18	.659/.567	.669/.645
21	.596/.518	.574/.551
26	.627/.556	.669/.659
32	.574/.517	.571/.560
34	.718/.677	.624/.609
37	.741/.656	.685/.666
45	.734/.632	.636/.612
Lack of inner control	$\overline{x}=.657/.528$	$\overline{x}=.586/.564$
8	.786/.647	.717/.693
15	.663/.560	.558/.538
16	.534/.376	.453/.420
17	.595/.422	.559/.535
25	.705/.598	.663/.636
29	.757/.687	.702/.689
46	.533/.406	.452/.434
Difficulty with emotional expression	$\overline{x}=.710/.543$	$\overline{x}=.637/.617$
7	.671/.521	.628/.609
12	.619/.423	.479/.456
23	.707/.594	.670/.652
28	.788/.638	.784/.771
40	.765/.538	.626/.597
Difficulty with cognitive expression	$\overline{x}=.689/.580$	$\overline{x}=.596/.578$
6	.668/.507	.566/.545
36	.565/.440	.521/.504
38	.775/.669	.740/.723
41	.714/.604	.633/.614
42	.755/.715	.649/.640
43	.656/.541	.539/.515
44	.689/.586	.525/.506
Oddness	$\overline{x}=.765/.508$	$\overline{x}=.570/.557$
11	.802/.606	.593/.578
19	.809/.697	.606/.595
39	.683/.222	.512/.498
Fear of losing control	$\overline{x}=.680/.359$	$\overline{x}=.545/.531$
20	.615/.361	.419/.401
30	.800/.463	.651/.635
33	.626/.254	.566/557
Restrained hostility	$\overline{x}=.644/.247$	$\overline{x}=.469/.449$
22	.669/.292	.520/.505
24	.758/.243	.505/.478
27	.505/.207	.381/.363
Automatisms	$\overline{x}=.782/.557$	$\overline{x}=.564/.548$
13	.784/.495	.541/.518
14	.879/.674	.560/.541
31	.683/.503	.592/.585
Favorable Scale (CSCV-F)		
Factor	Item – factor correlation	Item – scale correlation
Item number	r_s_	r_s_
Vital satisfaction	$\overline{x}=.737/.588$	$\overline{x}=.689/.623$
50	.757/.607	.668/.608
56	.726/.557	.698/.648
57	.770/.638	.756/.711
58	.697/.558	.623/.529
59	.737/.578	.688/.619
Self-esteem	$\overline{x}=.673/.401$	$\overline{x}=.590/.499$
47	.598/.318	.458/.359
48	.731/.439	.617/.510
49	.797/.596	.686/.604
51	.565/.250	.599/.523
Harmony	$\overline{x}=.748/.535$	$\overline{x}=.661/.593$
52	.754/.495	.682/.616
53	.710/.512	.580/.512
54	.691/.425	.582/.495
55	.835/.707	.798/.747

With N = 61, if r_s_ ≥ 0.247, *p* ≤ 0.05; if r_s_ ≥ 0.311, *p* ≤ 0.01; if r_s_ ≥ 0.398, *p* ≤ 0.001

### Statistical analyses

Using the SPSS v.20 statistical package, a series of analyses were run: 1) sample size adequacy (Kaiser-Meyer-Olkin [KMO] index) and data normal distribution (Kolmogorov-Smirnov test, skewness and kurtosis) for each scale; 2) correlations between each item with its corresponding mean factor score and mean scale score; 3) internal consistency of each scale and factor with Cronbach’s α, and with scale-factor correlations; and 4) construct validity, estimating the underlying factors with an exploratory principal component analysis with orthogonal varimax rotation, retaining factors agreeing with Kaiser’s criterion (a.k.a. root latent criterion) (eigenvalue > 1) and items with at least a 0.40 communality value.

## Results

### Sample size adequacy and normal distribution of data

Kaiser-Meyer-Olkin (KMO) values for sample adequacy were 0.55 for CSCV-D and 0.85 for CSCV-F. KMO statistic varies between 0 (indicating a disperse pattern of correlations; hence, factor analysis would be inappropriate) and 1 (indicating correlations have a rather compressed pattern; thus, factor analysis should yield reliable and definite factors). A minimum of 0.5 is recommended; yet, the closer to 1 the more reliability can be presumed. Mean CSCV-D (D _(61)_ = 0.12, *p* ≤ .05) and CSCV-F (D _(61)_ = 0.18, *p* ≤ .001) scores were not normally distributed; kurtosis levels for CSCV-D (*z* = 0.0331) scores and CSCV-F (*z* = 0.8212) scores were not significant. Skewness was significant for both scales (CSCV-D, *z* = 2.91, *p* ≤ .01; CSCV-F, *z* = 2.28, *p* ≤ .05) since participants reported a largely favorable QoL (agreeing mostly with CSCV-F statements and largely disagreeing with CSCV-D statements). In response, four kinds of correlational analyses were run with the Spearman non-parametric test.

### Item-factor and item-scale correlations

All item-factor correlations were significant (*p* < 0.001), as were all item-scale correlations (*p* < 0.01). When using corrected scores (i.e. item 1 is correlated with the total score obtained with all of its corresponding items but item 1, and so on) correlation values decreased slightly but remained significant (*p* < 0.05), save for the correlations between items 39, 24, 27 and their corresponding factors (“oddness” and “restrained hostility”). Item-factor correlation values ranged from 0.21 to 0.72, and mean correlation by factor ranged from 0.25 to 0.60.

Item-scale correlation values ranged from 0.33 to 0.77 for CSCV-D and from 0.36 to 0.75 for CSCV-F. Mean item-scale correlations ranged from 0.47 to 0.64 (corrected, from 0.45 to 0.62) for CSCV-D factors and from 0.59 to 0.69 (corrected, from 0.50 to 0.62) for CSCV-F factors. All these correlation values fall within the moderate range (≥ 0.30 and < 0.70). Given that item-scale correlation values were not extreme (r_s_ ≤ 0.30 or r_s_ ≤ 0.90) and all were significant, all original items were included for the subsequent analyses (see summary, Table 1).

Bartlett’s test results were significant (*p* ≤ 0.001) for both scales and all twelve factors; therefore the items were assumed to be related. In addition, R-matrix determinant values for all factors and the CSCV-F scale ranged from 0.002 to 0.758, thus rejecting multicollinearity (< 0.00001). Given that the items were not excessively related, the factor analysis was continued.

### Internal consistency and factor-scale correlations

Alpha (α) values were above 0.80 in both the CSCV-D and CSCV-F scales, suggesting good internal consistency. All twelve factors had an α value above 0.50. CSCV-D α values ranged from 0.576 (“restrained hostility”) to 0.885 (“loss of energy”); mean = 0.728. CSCV-F α values ranged from 0.671 (“self-esteem”) to 0.802 (“vital satisfaction”); mean = 0.742 (Table 2).

**Table 2:** Internal consistency of scales and factors

	Number of items	α	Confidence interval		Number of items	α	Confidence interval
			95 %				95 %
CSCV-D	46	.962	.95–.98	CSCV-F	13	.897	.85–.93
Lack of cognitive apprehension	5	.664	.51–.78	Vital satisfaction	5	.802	.71–.87
Loss of energy	10	.885	.84–.92	Self-esteem	4	.671	.51–.79
Lack of inner control	7	.814	.73–.88	Harmony	4	.753	.63–.84
Difficulty with emotional expression	5	.781	.68–.86				
Difficulty with cognitive expression	7	.832	.76–.89				
Oddness	3	.710	.56–.82				
Fear of losing control	3	.577	.35–.73				
Restrained hostility	3	.576	.35–.73				
Automatisms	3	.710	.56–.82				

Correlation between the CSCV-D and CSCV-F was moderate (−0.57) but significant (*p* ≤ .001). Correlations below 0.80 were observed between CSCV-D and three of its factors: “restrained hostility” (0.69), “automatisms” (0.68) and “oddness” (0.72). The remaining six factors scored from 0.80 to 0.93. Correlations between CSCV-F and its corresponding factors ranged from 0.86 to 0.93. Correlations between the nine CSCV-D factors varied from 0.43 to 0.81, and between the three CSCV-F factors they ranged from 0.66 to 0.78. All correlations were significant (*p* ≤ 0.001). A very few significant correlations between a scale and factors of the opposite scale were observed, although the significance level was quite low: between CSCV-D and favorable factors, from −0.46 to–0.56; between CSCV-F and disfavorable factors, from −0.29 and −0.67; favorable and disfavorable factors, from −0.15 and −0.66.

### Factor analysis

After rotation, eleven factors met Kaiser’s criterion, explaining 77.4 % of the CSCV-D variance; the first factor explained 9.76 % and the following explained 9.53 %. For variance in the CSCV-F, two factors met the criterion after rotation, explaining 55.1 % of variance; the first factor explained 32.0 % and the next 23.1 %. Loading patterns did not clearly replicate the original nine factors of the CSCV-D or three factors of the CSCV-F (Table 3).

**Table 3:** Factor loadings for each item onto each factor after rotation

Disfavorable Scale (CSCV-D)											
Factor:	1	2	3	4	5	6	7	8	9	10	11
% of variance:	9.758	9.531	8.825	8.271	7.503	6.843	6.491	5.448	5.287	5.215	4.241
Lack of cognitive apprehension											
1						.819					
2						.638					
3						.700					
5									.442		
35	.435										.752
Loss of energy											
4					.743						
9	.659										
10	.602										
18								.427			
21					.527						
26		.469									
32	.504			.478							
34	.729										
37				.750							
45										.608	
Lack of inner control											
8	.546										
15		.481									
16							.822				
17			.471					.526			
25			.464	.400		.414					
29							.483				
46	.418			.447							
Difficulty with emotional expression											
7					.605						
12			.443								
23							.627				
28		.645									
40							.408				
Difficulty with cognitive expression											
6		.811									
36					.708						
38		.426									
41		.552								.437	
42		.701									
43										.623	
44										.753	
Oddness											
11			.769								
19			.726								
39								.811			
Fear of losing control											
20				.560					.515		
30	.423			.476							
33		.550									.498
Restrained hostility											
22				.404							.575
24			.586		.454						
27									.803		
Automatisms											
13				.685							
14			.649	.495							
31	.662										
Favorable Scale (CSCV-D)											
Factor:	1	2									
% of variance:	32.036	23.105									
Vital satisfaction											
50	.637										
56	.502	.523									
57	.604	.502									
58		.538									
59	.579										
Self-esteem											
47		.892									
48		.517									
49		.652									
51	.840										
Harmony											
52	.497	.438									
53	.757										
54	.642										
55	.708	.403									

*Note*: Only loadings ≥ .400 are reported

## Discussion

Development of the CSCV was aimed at meeting the need for a tool to assess subjective QoL from the perspective of schizophrenic patients. The present study expands on previous studies of the CSCV’s psychometric properties and replicates its adequacy in a sample of patients from Mexico. Analysis of the CSCV-D and CSCV-F scales showed that the items were more strongly correlated to their scale than to their corresponding factors; however, in almost all cases results were significant. Mean correlations by factor between each scale (CSCV-D and CSCV-F) and its items showed a similar pattern of moderate values.

Internal consistency was significant and satisfactory; it was notably higher in scales than in factors. These results and values are comparable to those previously reported for this instrument with samples from Spain (CSCV-D, α = 0.94; CSCV-F, α = 0.85) [15] and Chile (CSCV-D, α = 0.962; CSCV-F, α = 0.897) [17]. The pattern of factor α values is also comparable to a study done in Chile [17]: CSCV-D from 0.350 (“restrained hostility”) to 0.830 (“loss of energy”), mean = 0.648; CSCV-F from 0.697 (“harmony”) to 0.772 (“vital satisfaction”), mean = 0.732. Given the factor-scale correlations, the disfavorable and favorable scales are clearly differentiated.

Factor analysis results agree with the number of factors obtained in a sample from Chile [17] (CSCV-D: 11, CSCV-F: 2), but differ from those reported in the original study [15] (CSCV-D: 9, CSCV-F: 3). Total explained variance was low (CSCV-D: 77.40 %; CSCV-F: 55.14 %), but higher than reported in previous studies from Spain (CSCV-D: 51.11 %; CSCV-F: 51.32 %) [14] and Chile (CSCV-D: 62.9 %; CSCV-F: 53.5 %) [17].

The psychometric properties of the CSCV in the studied sample of Mexican outpatients with psychosis were effective when considering CSCV-D and CSCV-D scale scoring. Nevertheless, scoring by factors was somewhat problematic, mainly for the factor “restrained hostility”, with two (out of three) non-significant corrected item-factor correlations, a moderate corrected item-scale and a marginal α value. The factor “oddness” had one item (out of three) with a non-significant corrected item-factor correlation and a moderate corrected item-scale, but its α value remained strong. In contrast, the factor “fear of losing control” had moderate correlations with all three of its corresponding items, but a marginal α value. The number of factors and their item loading pattern did not correspond to those reported in the original paper [14], an inconvenience also reported in a study in Chile [17]. The instability of factor structure might have been influenced by the relatively small sample in this study, in comparison to the size of those from Spain (*n* = 236) [14], and Chile (*n* = 183) [17]. Although Chilean and Mexican samples yielded equal number of factors for both scales, further analysis comparing factors’ composition was not possible as the necessary data from the former was not available. The CSCV is potentially a very useful QoL assessment tool in psychotic patients, and produces satisfactory psychometric properties. However, the present results suggest that scale scoring is more appropriate than factor scoring in this instrument. Further research is needed to better assess its psychometric properties (particularly its factor construct validity) and its applicability in other (non-Spanish-speaking) populations.

Limiting factors in the present study include its small sample size and use of an exploratory principal component analysis, both of which restrict the ability to generalize from its findings. Also, the KMO values for sample adequacy were acceptable, but low. The results from this sample of Mexican patients with psychosis provide further evidence in favor of the CSCV as an effective QoL assessment instrument in psychotic patients. As with any other assessment tool, caution is needed when applying its specific psychometric features to different populations.

## Conclusions

In mental health patients, QoL is now acknowledged as an important factor. Assessing QoL helps in monitoring overall patient status and promotes better patient-staff communication. Patient opinion on their QoL also provides useful information for use in designing and improving clinical services. The CSCV for psychotic patients stands out as a useful tool for this purpose, particularly for mental health professionals in Spanish-speaking countries. The present results support the CSCV as a valid and reliable instrument, although interpretation is best done using scale scores rather than factor scores.

## Abbreviations

CSCV, Cuestionario Sevilla de Calidad de Vida/Seville Quality of Life Questionnaire; CSCV-D, Seville quality of life questionnaire disfavorable scale; CSCV-F, Seville quality of life questionnaire favorable scale; QoL, quality of life.
